# Effect of Pulse Electrodeposition Parameters on the Microstructure and Mechanical Properties of Ni–W/B Nanocomposite Coatings

**DOI:** 10.3390/nano12111871

**Published:** 2022-05-30

**Authors:** Malay Kumar Das, Santhosh Kumar J. Urumarudappa, Sarwar Kamal, Yoanda Widiadita, Arif Mahamud, Thiranan Inthawong Saito, Thiti Bovornratanaraks

**Affiliations:** 1 Extreme Conditions Physics Research Laboratory and Center of Excellence in Physics of Energy Materials, Department of Physics, Faculty of Science, Chulalongkorn University, Bangkok 10330, Thailand; malaykumardas90@gmail.com; 2 Department of Post Graduate Studies and Research in Biotechnology, Jnana Sahyadri, Kuvempu University, Shankaraghatta, Shimoga 577451, India; Santhu.ju@gmail.com; 3 School of Ecology and Conservation, Department of Crop Physiology, University of Agricultural Sciences, GKVK, Bangalore 560065, India; 4 Department of Logistics and Supply Chain Management, Dr. Sulaiman Al Habib Hospital, Riyadh 14212, Saudi Arabia; sarwarkamal2@gmail.com; 5 Faculty of Biomedical Sciences, University of Lampung, Bandar Lampung 35141, Indonesia; yoanda1190@gmail.com; 6 Department of Electrical and Computer Engineering, North South University, Dhaka 1229, Bangladesh; fmarif2009@gmail.com; 7 Faculty of Humanities, Bangkok University, Pathum Thani 12120, Thailand; thiranansaito29@gmail.com; 8 Thailand Center of Excellence in Physics, Commission on Higher Education, 328 Si Ayutthaya Road, Bangkok 10400, Thailand

**Keywords:** Ni–W/B nanocomposite coatings, pulse current electrodeposition, direct current electrodeposition, hardness, wear resistance

## Abstract

In this work, Ni–W/B nanocomposite coatings were successfully fabricated on low carbon steel by using pulse current (PC) electrodeposition. The effects of the frequency and duty cycle on the microstructure, wear resistance, and microhardness of the coatings were studied. The results obtained show that the distribution and content of boron particles (>4 wt.%) in the PC electrodeposition coatings are significantly better than those of direct current (DC) electrodeposition coatings (less than 4 wt.%). The hardness results reveal that the highest microhardness of 1122 HV can be obtained at a frequency of 100 Hz and duty cycle of 30%. Furthermore, the relationship between the microstructure and mechanical properties was discussed.

## 1. Introduction

Electrodeposition of Ni–M alloys, where M can be W, Co, Fe, B, P, etc., has been studied widely and reported to have improved properties compared to pure Ni [1,2,3]. Mechanical and chemical properties such as hardness, wear resistance, self-lubrication, and corrosion resistance can be further improved by the incorporation of particles during the electrodeposition of metals and alloy coatings. For example, previous studies demonstrate that the hardness, wear resistance, and corrosion resistance can be further increased by adding reinforcing particles (i.e., boron, diamond, SiC, and Al_2_O_3_) into nickel-based alloys [4,5,6,7,8,9,10,11,12,13,14,15].

In our previous work, we reported that Ni–W/B composite coatings were fabricated by a direct current (DC) electrodeposition method using a Ni–W electrodeposition bath containing different amorphous boron concentrations [14]. The results show that the hardness and wear resistance can be enhanced by the incorporation of boron in Ni–W coatings [14]. In addition, the reported results also demonstrate that the microstructure and mechanical properties of composite coatings can be further increased by changing DC to pulse current (PC) electrodeposition. The pulse parameters (e.g., duty cycle, frequency) can strongly affect the distribution and contents of the reinforcing particles in the coatings [7,10,13]. Chen et al. [7] reported that the pulse frequency can change the microstructure, wear resistance and preferred orientation of electrodeposited Ni–Al_2_O_3_ composite coatings. Goldasteh et al. [10] studied the effect of pulse plating parameters on the microstructure and mechanical properties of Ni–W–TiO_2_ nanocomposite coatings. Their results indicated that the crystalline size can be reduced by PC electrodeposition, resulting in an enhancement of the hardness of the coating. Lajevardi et al. [13] reported the influences of pulse electrodeposition parameters on the properties of Ni–TiO_2_ nanocomposite coatings. The Ni–TiO_2_ coatings prepared by pulse current electrodeposition appear to contain more incorporated TiO_2_ and higher microhardness. Moreover, Low et al. [16] reported that pulse current can increase the content of incorporated nanoparticles and enable a wider range of deposit compositions and properties.

It is noteworthy that, as mentioned above, there are many studies concerning the effect of pulse parameters on the microstructure and mechanical properties of composite coatings. However, the effect of PC electrodeposition of Ni–W/B nanocomposite coatings and the influences of pulse parameters on them are still not reported. Therefore, in this paper, Ni–W/B nanocomposite coatings are fabricated by PC electrodeposition. The effect of pulse frequency and duty cycle on the amount of incorporated boron particles and their morphology are studied. Furthermore, the microhardness and wear resistance of Ni–W/B composite coatings were evaluated. Moreover, the mechanism of the microstructure and mechanical properties has been discussed.

## 2. Experimental

**Figure 1:**
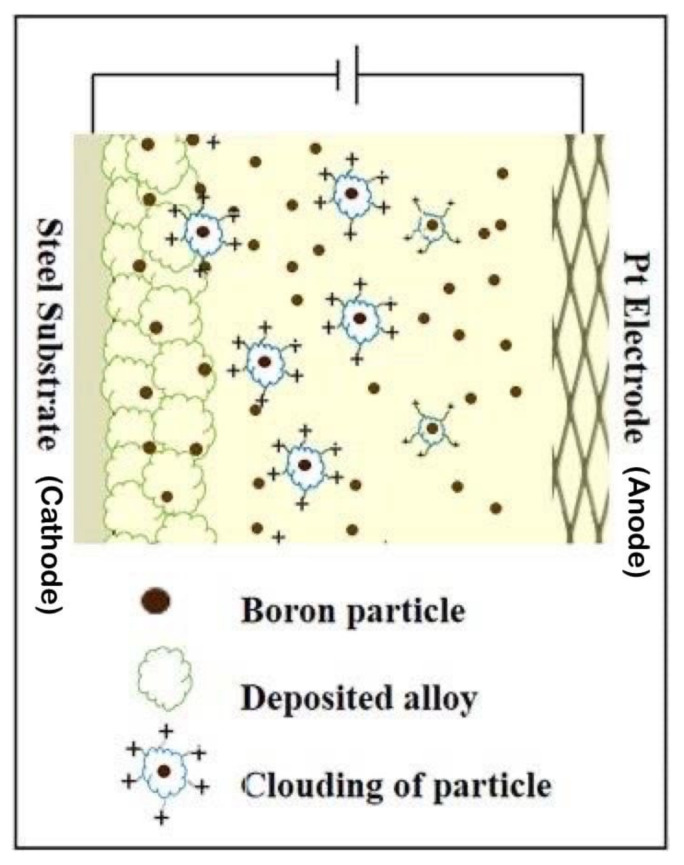
Schematic representation of the boron co-deposition on the base Ni–W alloy matrix.

In this work, PC electrodeposition was applied to prepare Ni–W/B nanocomposite coatings. The electrodeposition bath and parameters are shown in Table 1. Amorphous boron particles were used during the electrodeposition process. The boron particles had an average diameter of 0.14 μm. The boron particles and other chemicls were obtained from Permata chemical distributors (Indonesia). Boron was dispersed in the plating bath by means of a magnetic stirrer which was set at a RPM of 150 throughout the deposition process. Boron particles were incorporated in the Ni–W alloy matrix by means of co-deposition as a composite particle into the alloy matrix to generate Ni–W/boron composite coatings. A schematic diagram for the co-deposition of the boron particles has been shown in Figure 1.

The steel plate was degreased by alkaline sodium hydroxide solution at 60 °C for 10 min, soaked in 14% hydrochloric acid at room temperature for another 10 min, and then rinsed in distilled water. During the electroplating process, the electrolyte temperature was controlled at 75 °C and stirred at 150 rpm by a magnetic stirrer.

### 2.1. Sample Preparation

The composite coatings were fabricated on a commercial purity low carbon steel substrate with a size of 2.0 cm × 2.0 cm. Platinum mesh (12.0 cm × 6.0 cm) was used as the anode, with approximately 3.5 cm spacing between the two electrodes. All chemicals used in the experiment were laboratory grade. Before electrodeposition, the plating bath with suspended boron particles was stirred at 300 rpm for 30 min and then sonicated for another 30 min to create well-dispersed particles.

### 2.2. Characterization

#### 2.2.1. Pulse Current Power Source

The pulsed current was applied to the system by a pulse rectifier (Dynatronic, Salt Lake, UT, USA, DUPR 10-3-6) under square wave periodic pulse conditions. Pulse frequencies of 40 Hz, 60 Hz, 80 Hz, and 100 Hz and duty cycles of 30%, 50%, and 70% were studied in this work. For comparison, Ni–W/B composite coatings were also prepared by direct current electrodeposition with a current density of 0.1 A/cm^2^.

#### 2.2.2. X-ray Diffraction (XRD) Analysis

The phase of the Ni–W/B composite coatings was studied by X-ray diffraction (Bruker AXS, Billerica, MA, USA), with a Cu target, K_α_, at a scan rate of 0.02°/min and a scanning angle in the range of 10–80°. Scherrer’s equation was used to calculate the grain size of the samples before and after heat treatment. Scherrer’s equation can be represented by the following formula:

(1) $$D=\frac{0.9\;\lambda }{\beta cos\;\theta }$$

In Equation (1), D is the grain size

*λ* is the X-ray wavelength (1.5418 Å)

*β* is the corrected peak width at half maximum intensity (FWHM) and *θ* is the Bragg angle.

#### 2.2.3. Scanning Electron Microscopy (SEM) Analysis

The samples were characterized and investigated in terms of morphology and microstructure by a Horiba scanning electron microscope (SEM) with energy-dispersive X-ray spectroscopy (EDS) capability.

#### 2.2.4. Cross-Section Preparation

Certain samples were cut into 1∗2 cm^2^ pieces for cross-sectional study using a low-speed saw and mounted in tiny molds filled with epoxy resins. The samples were ground and polished by a Buehler Ecomet 250 automated grinder/polisher.

#### 2.2.5. Hardness

The microhardness of the NWB deposits were measured on the surface using Mitutoyo hardness tester with a Vickers’s diamond indenter under a load of 100 g (0.98 N) at seven different locations of the coating. The dwell time for each indentation was 15 s. The Vickers hardness can be calculated in accordance with the formula:

(2) $$H_{v}=\frac{1854}{Ld^{2}}$$

In Equation (2), *H_v_* is the hardness in Vickers’s and

*L* is the applied load and

*d* is the diagonal of the indentation.

The average value of the five measurements (except the maximum and minimum values) has been reported as the hardness of the composite coating obtained.

#### 2.2.6. Wear

The tribological property of the deposits was investigated by a means of a wear resistance test. The wear test was conducted at an air humidity of 55 ± 5 RH% and a temperature of 25–30 °C using a ball-on-disc tribometer with the sample placed horizontally on a turntable. A zirconium dioxide ball was used as a counter-material. The hardness of the zirconium dioxide ball was ~1300 *H_v_*. Before each test, both the sample and the ball counter face were ultrasonically cleaned in acetone for 10 min, and dried by hot air. The anti-wear performance of the films was estimated from the weight loss of the specimens The tests were performed by applying a load of 10 N to a zirconium dioxide ball of diameter 6 mm, speed of 300 rpm, track radius of 5.0 mm for a total sliding distance of 800 m.

#### 2.2.7. Inductively Coupled Plasma–Mass Spectrometry (ICP-MS) Analysis

The boron content in the coatings was evaluated by inductively coupled plasma–mass spectrometry (ICP–MS).

ICP-MS analysis stands for inductively coupled plasma-mass spectrometry. It involves ionization of the test sample under consideration by an extremely hot plasma which is usually made up of by argon gas. During the analysis process sample ions are taken out through several cones which are then subsequently passed into a mass spectrometer. The ions are then separated by mass-to-charge ratio which is then transmitted to an end detector which reports the ion signal relative to the concentration of the element under consideration.

## 3. Results and Discussion

**Figure 2:**
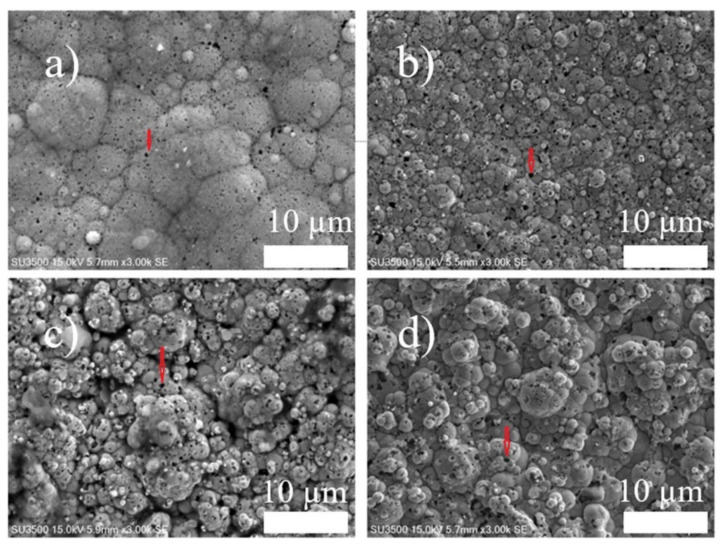
Scanning electron microscopy (SEM) images of Ni–W/B coatings prepared at a boron concentration of 10 g/L, peak current density of 0.20 A/cm^2^, frequency of 100 Hz and different duty cycles: (**a**) 30%, (**b**) 50%, (**c**) 70%, and (**d**) direct current (DC).

Figure 2a–c shows the SEM images of the coatings under different duty cycles. For comparison, SEM images of the coatings prepared by DC electrodeposition are also shown in Figure 2d. In Figure 2a–c, it can be seen that the columns exhibit cauliflower shapes of different sizes, and the coatings prepared at a duty cycle of 30% show flattened and extended columns. The black points (arrow point) are the embedded boron particles. SEM can indicate that the Ni–W/B composite coatings are successfully fabricated on low carbon steel substrates by both PC and DC electrodeposition.

**Figure 3:**
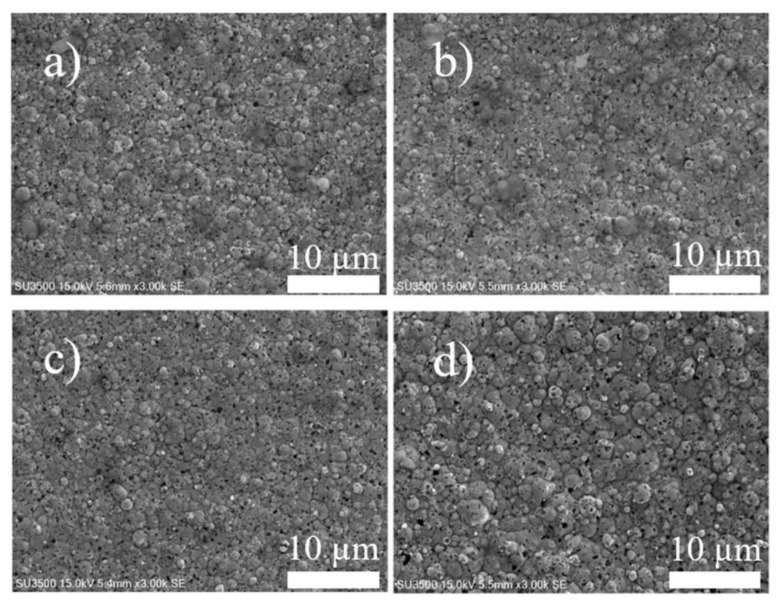
SEM images of Ni–W/B coatings electrodeposited at a boron concentration of 10 g/L, peak current density of 0.20 A/cm^2^, 50% duty cycle and different frequencies: (**a**) 40 Hz, (**b**) 60 Hz, (**c**) 80 Hz, and (**d**) 100 Hz.

In addition, the effect of the frequency on the microstructure was also studied. SEM micrographs of the coatings prepared at a duty cycle of 50% and various frequencies are shown in Figure 3a–d. Embedded boron particles can be seen as dark spots on the gray background of the cauliflower-shaped Ni–W matrix. The SEM images reveal that the composite coatings prepared by PC electrodeposition have fine and dense microstructures and small uniformly distributed columns. The composite coatings prepared by DC electrodeposition exhibit coarser surfaces, fewer embedded borons, and larger columns.

**Figure 4:**
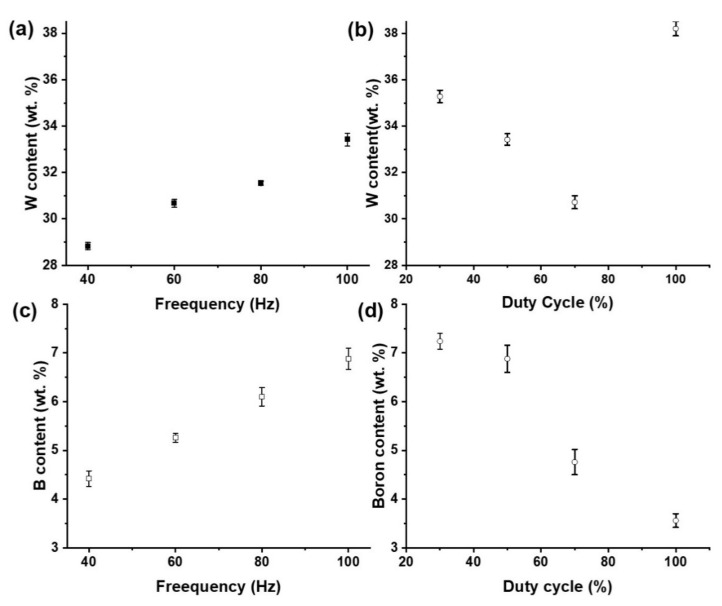
Tungsten content in the coatings, (**a**) varying by frequency at 50% duty cycle, (**b**) varying by duty cycle at frequency of 100 Hz. Boron content in the coatings, (**c**) varying by frequency at 50% duty cycle, (**d**) varying by duty cycle at frequency of 100 Hz.

**Figure 5:**
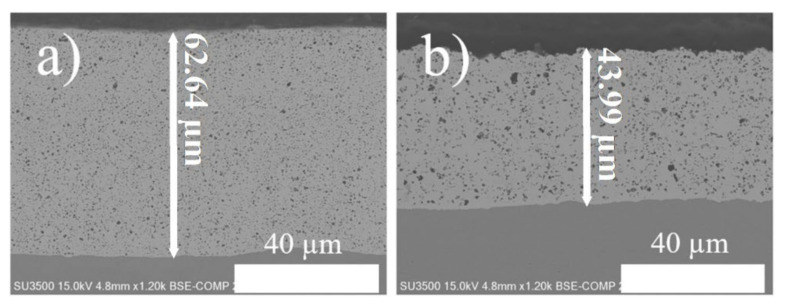
Cross-sectional SEM images of Ni–W/B coatings (**a**) prepared by pulse current with a peak current density of 0.2 A/cm^2^, frequency of 100 Hz, and 50% duty cycle, (**b**) prepared by direct current with a current density of 0.1 A/cm^2^.

The tungsten content in the deposits was determined by EDS, as shown in Figure 4a,b. The tungsten in the coatings increases with increasing frequency (Figure 4a), while it decreases with increasing duty cycle (Figure 4b). However, the tungsten content in all coatings prepared with pulse current deposition is lower than that of direct current deposition. The variation of duty cycle certainly affects the boron, the W content of the deposits. The sample fabricated with the direct current showed the highest W content among all samples while returning the lowest value of B content. The samples fabricated at 30% duty cycle showed the highest amount of B content in the deposits. The introduction of pulse current parameters, therefore, evidently affects the dispersion of B particles in the deposits (Figure 5). This is due to the near-linear enhancement of the co-deposition rate of boron particles and in turn the boron content in the deposits (Figure 4) on the base Ni–W matrix as the duty cycle is reduced from 100% to 30%. Co-deposition of boron particles certainly seems to be more favorable as the duty-cycle is systematically altered to 30%, thereby causing the effective current density during electrodeposition to attain lower values as compared to the samples fabricated at DC current with 100% duty cycle. It becomes evident that the boron content of the deposits (Figure 4) and in turn the actual dispersion of boron particles in the deposits (Figure 5) directly depends on the effective current density during the electrodeposition process.

The frequency of the power supply also significantly affects the boron content, W content in the deposits as both the W content and B content tend to become enhanced in an almost a linear variation (Figure 4). The increase in the power frequency during the deposition process enhances the co-deposition rate and subsequently the content of W-boron particles into the deposits. This in turn also directly affects the dispersion of boron particles in the deposits as compared to the NWB samples fabricated under direct current (Figure 5).

To evaluate the boron content in the coatings, ICP analysis was performed for the nanocomposite coatings prepared at different duty cycles and frequencies. The ICP results (Figure 4c,d) show that the embedded boron particles increased from 3.56% when applied by direct current to as high as 7.24% when applied by pulse current. Similar results can be observed from the cross-sectional images in Figure 4a,b. In the comparison of Ni–W/B prepared by direct current (Figure 5a) and by pulse current (Figure 5b), the coating applied by rectangular waveform pulse current with a 50% duty cycle has a significantly higher number of visible embedded boron particles than that of the sample prepared by direct current. The size of the co-deposited particles appear to be significantly varying for the samples prepared by DC as compared to the sample prepared by pulse plating with a duty cycle of 30%. Moreover, each embedded particle appears to be smaller for coatings prepared by PC electrodeposition. This is due to the effect of differences in relaxation time (off-time), which enhances the nucleation rate. The samples fabricated with PC deposition tend to attain a higher level of clouding of boron particles on the base Ni–W matrix because of the significant absence of the plating current (off-time) the nucleation sites tend to get more agglomerated with the neutral boron particles which also enhances the density of dispersion of the boron particles along with a refinement of the size of the co-deposited particles on the base Ni–W matrix. Figure 2a shows that the Ni–W/B coating applied by a 30% duty cycle, which has a longer ‘off-time’ than other conditions, has a different morphology compared to other conditions. The surface appears to be very flat and smooth due to the strong grain refinement effect.

Figure 5 shows that the Ni–W/B composite coating created by PC electrodeposition has a much thicker thickness than the composite coating prepared by DC electrodeposition. One can see that the embedded boron particles formed were uniformly distributed in the Ni–W matrix for the coating fabricated by PC electrodeposition. The number, shape, size, and distribution of reinforcing embedded boron particles significantly affect the microhardness and wear resistance of the composite coating. From the cross-sectional view (Figure 5) it is evident that the boron particles are deposited as standalone particles which are clearly distinguishable from the base Ni–W alloy matrix. The co-deposited boron particles appear to be more uniformly dispersed throughout the base alloy (Ni–W) matrix for the PC plated sample as compared to the DC plated sample. From previous research [17], Ni crystals prefer to grow into large columns if there is no inhibition by the second phase of embedded particles. Therefore, the embedded boron particles do inhibit crystal growth, resulting in small, dense, and flat columns in the prepared Ni–W/B composite coatings. The co-deposited boron particles appear to be more uniformly dispersed throughout the base alloy (Ni–W) matrix for the PC plated sample as compared to the DC plated sample (Figure 5). The sample fabricated at 50% duty cycle reveals a more uniform dispersion of boron particles through the base matrix than the sample fabricated in DC plating conditions. Therefore, this could be the reason that the grain size of the samples fabricated in PC plating conditions tend to report a smaller grain size than the samples fabricated in DC plating conditions. The grain size seems to depend on the B content and the density of dispersion of the co-deposited boron particles on the base matrix. This is a direct consequence of the grain growth inhibition caused by the embedded composite boron particles. Figure 7a also shows marginal decline in the grain size upon increasing the frequency of deposition as the boron content of the coating is increased upon increasing the frequency in Figure 4c which is similar to the increasing trend of grain size observed in Figure 7b upon an increase of duty cycle, as the boron content of the deposits also progressively declines upon an increase of the duty cycle (Figure 4d). It can also be observed from the schematic representation of the deposition process in Figure 1 that the boron particles tend to become embedded between the grain boundaries and, thereby, it can be expected that a denser agglomeration or uniform distribution of the embedded boron particles would inhibit the natural growth of the grain boundaries, limiting the grain growth and in turn causing a relative reduction in the grain size of the deposits.

**Figure 6:**
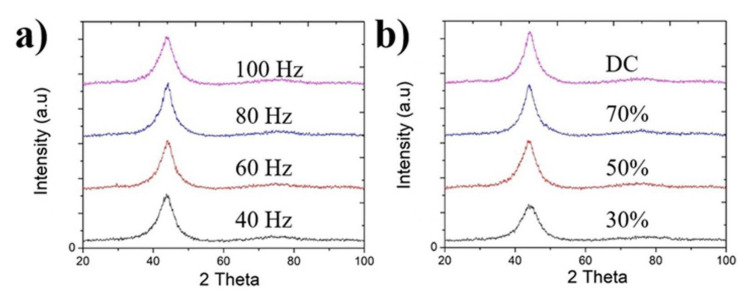
X-ray diffraction (XRD) patterns of coatings with (**a**) varying frequency from 40–100 Hz at 50% duty cycle, (**b**) duty cycle from 30–70% at frequency of 100 Hz and direct current.

**Figure 7:**
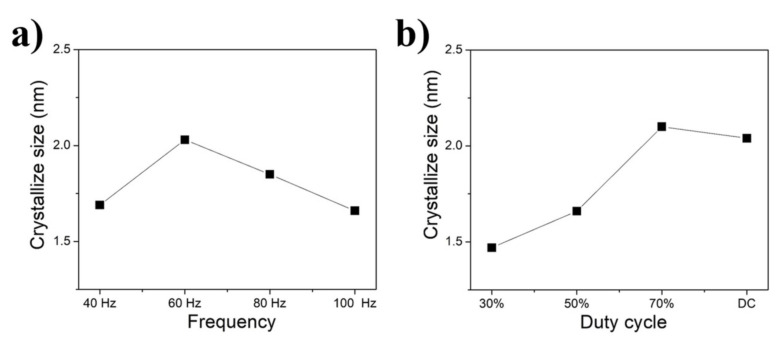
Effect of frequency and duty cycle on the crystallite size of the coatings (**a**) varying by frequency at 50% duty cycle, (**b**) varying by duty cycle at frequency of 100 Hz.

The crystallite sizes estimated by XRD are presented in Figure 6. XRD patterns show the main phase to be Ni(111). Using the width of the Ni(111) peaks, the crystallite size was calculated by the Scherrer equation and is illustrated in Figure 7. PC electrodeposition can further decrease the crystallite size of the coatings. For DC electrodeposition with a boron concentration of 10 g/L, the crystallite size was 2.1 nm (Figure 7b). By changing to pulse current, the crystallite size is significantly reduced to as low as 1.5 nm for the nanocomposite coating prepared at 100 Hz frequency and 30% duty cycle (Figure 7b). This event is also the effect of incorporated boron nanoparticles acting as grain refiners, which are heterogeneous nucleation spots in the Ni–W matrix. A similar result was obtained in Ni–B coating experiments [8]. Figure 7b shows that the crystallite size decreases as the duty cycle decreases. To maintain the same average current density as the duty cycle decreases, the peak current density will be increased accordingly. Increasing the peak current density leads to an excessive electrical potential, which also enhances the nucleation rate and forms very fine crystals. As the duty cycle decreases, ‘off-time’ increases and ‘on-time’ decreases. Studies have shown that a longer ‘off-time’ may provide more chances for free-moving particles in the electrolyte to reach the cathode and form a double layer on the coating [6,13]. This conclusion is in good agreement with Chandrasekar’s experiment [18]. A longer ‘off-time’ provides a chance for mass transportation; during the ‘on-time’ area around the cathode, the current density accumulates, hence leading to overactivation of the electrochemical process and turning into an area with insufficient ions for an effective continuous process. Applied by a longer ‘off-time’, the stirred solution will have a longer time to convey the masses of needed ions to the cathode. Hence, the overall electrolyte will have a more uniform distribution of ions, which is necessary for the high-quality electrodeposition process with more incorporated particles [19]. Furthermore, Figure 5 shows that smaller incorporated boron particles are preferable for high thickness coatings than large boron particles. As mentioned previously, a longer ‘off-time’ enhances the mass conveying and mass distribution process, which means that the stirred electrolyte will carry miniature boron particles to the vicinity of the cathode surface. Many miniature boron particles will become trapped inside the forming Ni–W matrix, which means an increase in the cathode thickness and cathode area. A thicker cathode allows more free-moving boron particles to become trapped and ends up with numerous nucleation sites forming very fine crystals; hence, the incorporated boron particle mass will accelerate the coating process.

The pulse frequency also plays a major role in the movement and deposition of the reinforcing particles [10]. The pulse frequency controls the total time for one cycle and the total number of cycles in the whole electrodeposition process. For the higher frequency, the total time for one cycle decreases, which means that both ‘on-time’ and ‘off-time’ are shorter. Shorter ‘on-time’ and ‘off-time’ means that boron particles are constantly disturbed. Hence, the particles have less chance to form larger clusters and embed small boron particles into the Ni–W matrix. These small dispersed boron clusters later become nucleation spots and hence decrease the crystallite size. In another way, the relatively larger boron particles would have less chance to incorporate into the Ni–W matrix, because the high pulse frequency reduces the deposition time (on-time) duration, hence, only relatively smaller boron particles would have a higher chance to co-deposit in the Ni–W layers. Small boron particles can be incorporated into the coating layers (Figure 7a), and the crystallite size decreases as the frequency increases from 60 Hz to 100 Hz.

**Figure 8:**
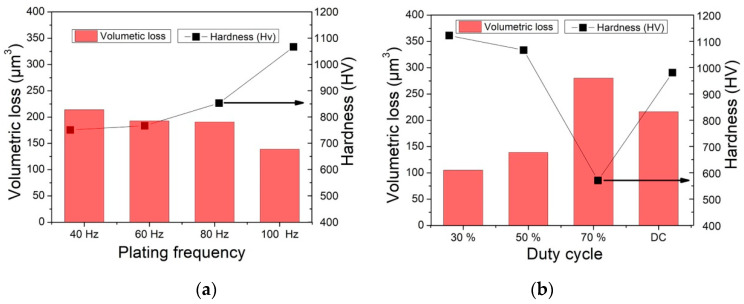
Effect of pulse frequency and duty cycle on the hardness and wear resistance, (**a**) varying by frequency at 50% duty cycle, (**b**) varying by duty cycle at frequency of 100 Hz.

The microhardness and wear resistance of the Ni–W/B nanocomposite coatings were investigated by a Vickers hardness testing machine and ball-on-disk tribometer. The microhardness and volumetric loss of the nanocomposite coatings prepared by both PC and DC electrodeposition are shown in Figure 8a,b. Figure 8 shows that the high microhardness of the coatings led to superior wear resistance. This conclusion is in good agreement with Archard’s equation, in which the total volume of worn-off particles produced (*Q*) is given by:

(3) $$Q=\frac{KWL}{H}$$

Where *K* is a constant whose dimension is not considered, *W* is the total normal applied force on the specimen, *L* is the sliding distance, and H is the hardness of the softest scathing surfaces. It is given from Equation (3) that the hardness has an inverse correlation with the volumetric loss of the coatings. A lower friction coefficient will decrease the values of variables *K* and *Q* in the equation and clarify the relationship between the friction coefficients and wear resistance.

Figure 8 demonstrates that the pulse frequency and duty cycle have strong effects on the microhardness of Ni–W/B composite coatings. The hardness of composite coatings is determined by the hardness of the metal matrix alloy and the content of codeposited particles. The hardness of metal matrix alloys can be influenced by the microstructure of the coatings, such as the alloy composition and grain size. As the frequency and duty cycle vary, the crystallite size changes between 1.5–2.2 (Figure 7). The slight difference in crystallite size does not affect the hardness too much, while the tungsten content increases to 33.42 wt.% from 28.83 wt.% (Figure 4a). The enhancement of the hardness of Ni–W alloy coatings with increasing tungsten content was already demonstrated by a previous report [1]. In addition, the boron content also increases with increasing frequency (Figure 4c). Therefore, the improvement in hardness for Ni–W/B composite coatings can be caused by both the Ni–W alloy and the incorporated boron particles. The influence of the duty cycle on the hardness can also be contributed by the tungsten and boron content in the composite coatings (Figure 3b,d). However, the high tungsten content and low boron content of coatings fabricated with DC deposition contribute to a low hardness. A lower incorporated boron content leads to lower dispersive strengthening [20] and a lower particle strengthening effect. This result reveals that the incorporated boron content may be the dominant factor for the hardness of Ni–W/B composite coatings.

**Figure 9:**
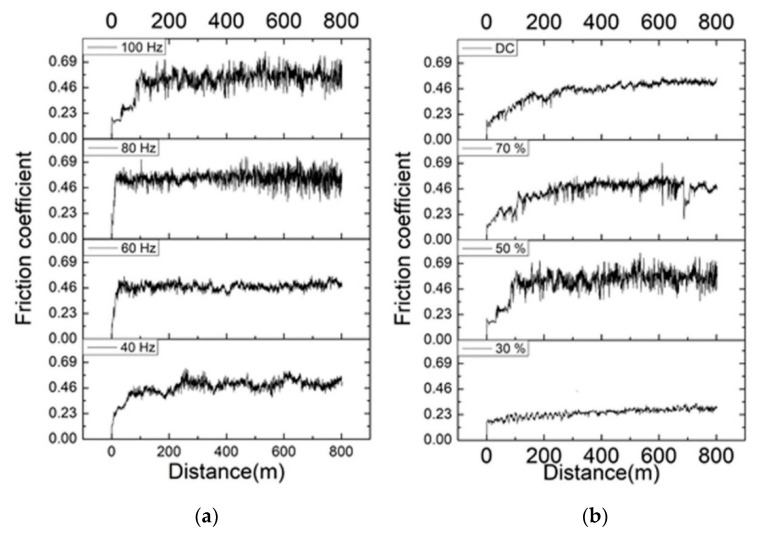
Friction coefficient of the coatings, (**a**) effect of frequency, (**b**) effect of duty cycle.

Figure 8 also demonstrates a significant correlation between the hardness and volumetric loss of the Ni–W/B composite coatings. The results indicate that the wear resistance is a linear function of the hardness for the deposited Ni–W/B composite coatings. In addition, the friction coefficient is also an important factor. In Figure 9b, the Ni–W/B composite coating prepared by a 30% duty cycle exhibits the lowest friction coefficients. This is the result of the difference in surface morphology being flat and smooth (Figure 3a), as mentioned previously. The Ni–W/B composite coating prepared by 100 Hz frequency and 30% duty cycle shows outstanding mechanical properties with a high hardness of 1122 HV (Figure 8b) and a low kinetic friction coefficient of 0.25 (Figure 9b). These two properties contribute to the distinctively high wear resistance performance (Figure 9b). Ni–W/B coatings have a significantly higher microhardness than those of Ni–W/BN, which achieves a maximum hardness of less than 580 HV [21]. In addition to Ni–W/BN composite coatings, Ni–W/B composite coatings are harder than Ni-W/SiC coatings (725 HV) [22]. Moreover, the incorporated boron particles of Ni–W coatings are also higher than those of Ni–W/diamond, and even composite coatings can easily exceed the microhardness of Ni–W/diamond composite coatings (~800 HV) [23]. In addition, the average kinetic friction coefficient of Ni–W/B composite coatings can reach ~0.25, which is lower than that of Ni–W/diamond composite coatings (~0.4). Due to its high hardness and low average kinetic friction coefficient, Ni–W/B composite coatings have shown a great wear resistance performance compared to other Ni–W-based composite coatings.

**Figure 10:**
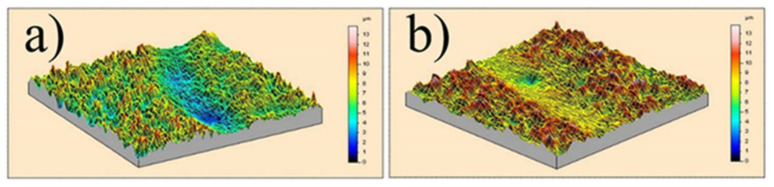
Section of the wear track profile of the Ni–W/B coating prepared at a frequency of 100 Hz and different duty cycles: (**a**) 70% and (**b**) 30%.

Figure 10 shows the wear track profile of nanocomposite coatings prepared at 100 Hz frequency and 70% duty cycle (Figure 10a) and 100 Hz frequency and 30% duty cycle (Figure 10b). From this 3D wear track profile, it can be seen that, presented on the same scale of height and deepness, the Ni–W/B coating prepared by a 70% duty cycle appears to have contrasting shades of colors compared to the image of the coating prepared by a 30% duty cycle, which has most of the surface area in similar shades of colors. The profile scan data show that the volumetric loss of nanocomposite coatings prepared at a 70% duty cycle is much larger than that of nanocomposite coatings prepared at a 30% duty cycle. This observation is in good agreement with the previously mentioned results.

Although many factors contribute to the wear resistance property of materials [23], hardness remains one of the most important factors. As shown in Figure 8a,b, the hardness plays a major role in predicting the wear resistance property of the Ni–W/B composite coating. Another factor is the friction coefficient. For the 30% duty cycle coating, it has a significantly lower friction coefficient than the other coatings. This is strongly related to its eminent wear resistance property.

## 4. Conclusions

Ni–W/B nanocomposite coatings were successfully fabricated by the PC electrodeposition method. The results obtained demonstrate that the pulse frequency and duty cycle have a strong influence on the morphology, crystalline size, boron content, microhardness, and wear resistance of nanocomposite coatings. Outstanding hardness and wear resistance can be obtained at 100 Hz frequency and 30% duty cycle. The coatings prepared with these pulse parameters exhibited the smallest crystallite size of 1.5 nm, low friction coefficient of 0.25, and highest hardness of 1122 Hv. The high wear resistance could be mainly caused by the high hardness, low friction coefficient, and high boron contents in the Ni–W/B nanocomposite coatings.

## Figures and Tables

**Table 1:** Basic bath compositions and electrodeposition conditions.

Composition and Conditions	
${\mathrm{NiSO}}_{4}$$\cdot 6H_{2}$O (g L^−^^1^)	18
${\mathrm{Na}}_{2}{\mathrm{WO}}_{4}\cdot 2H_{2}O$ (g L^−1^))	53
${\mathrm{Na}}_{3}C_{6}H_{5}O_{7}\cdot 2H_{2}O$ (g L^−1^)	168
${\mathrm{NH}}_{4}\mathrm{Cl}$ (g L^−1^))	31
NaBr (g L^−1^)	18
Boron particle (g L^−1^)	10
Temperature (°C)	75
pH	8.10–8.86
Peak current density (A ${\mathrm{cm}}^{-2}$)	0.2
Pulse duty cycle	30%, 50%, 70%
Pulse frequency (Hz)	40, 60, 80, 100

## Data Availability

The datasets generated during the current work are available from the corresponding author on reasonable request.
